# Combined Hepatocellular Carcinoma and Fibrolamellar Carcinoma Presenting as Two Adjacent Separate Lesions in a Young Boy: First Case Report from Asia

**DOI:** 10.1155/2013/101862

**Published:** 2013-03-03

**Authors:** Pradyumn Singh, Banumathi Ramakrishna

**Affiliations:** Department of Pathology, Christian Medical College, Vellore, Tamil Nadu 632004, India

## Abstract

We report a rare case of combined hepatocellular carcinoma and fibrolamellar carcinoma arising in a noncirrhotic liver, in a 14-year-old boy who underwent right hepatectomy. We discuss the clinicopathological and immunohistochemical features and the clinical outcome in this unusual tumor.

## 1. Introduction

Hepatocellular carcinoma of adult type (HCC) and fibrolamellar carcinoma (FLC) are two distinct entities unique in their clinical, histological, and biological aspects [1]. There have been occasional reports of combined occurrence of FLC and HCC [2–5]. However, as the criteria to diagnose combined occurrence is not yet established in the literature, there seem to be cases which in a true sense are not combined occurrence of FLC-HCC but rather an admixture of FLC-like areas in the usual HCC thus confusing the literature. Here, we report a rare case of a separate FLC and HCC presenting synchronously in a 14-year-old boy, with well-characterized morphology and treatment outcome, and discuss the distinctive features which would help in the correct identification of such unusual lesions. 

## 2. Case Report

A 14-year-old boy presented with history of right sided abdominal pain for 2 months. On examination there was an irregular, firm, swelling palpable in the liver extending up to 7 cm below the right costal margin. Computed tomography (CT) of the abdomen showed a large 9.6 × 10.2 × 10.5 cm well-defined, heterogeneously enhancing mass in the right lobe of liver in segments 6 and 5 and another lobulated 9.8 × 6.7 cm mass with homogenous enhancement in segments 8 and 7 suggestive of malignant liver masses with intrahepatic metastases. Investigations showed no elevation of serum alphafetoprotein (AFP), beta human chorionic gonadotrophin, or carcinoembryonic antigen. Hepatitis B surface antigen, hepatitis C antibody, and human immunodeficiency virus antibody were negative. Liver function tests at the time of admission showed total serum bilirubin level of 0.4 mg/dL, direct 0.2 mg/dL, total protein 8.2 g/dL, albumin 3.8 g/dL, aspartate aminotransferase (AST) 289 U/L, alanine aminotransferase (ALT) 99 U/L and alkaline phosphatase (ALP) 143 U/L, and INR of 0.96. There was no history of parasitic infestation or known exposure to environmental toxins. A wedge biopsy from the liver mass revealed a moderately differentiated hepatocellular carcinoma following which the patient underwent right hepatectomy.

**Figure 1:**
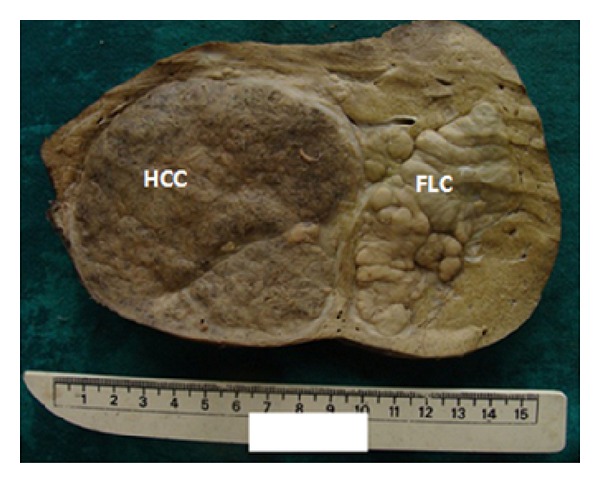
A case of combined HCC (brown cut surface) and FLC (lobulated grey white cut surface)—right hepatectomy specimen.

Histopathological findings: grossly, the right lobe of the liver weighed 1000 g. The external surface was focally nodular and on sectioning revealed a circumscribed tumor measuring 10 × 8 × 12 cm with solid brown cut surface almost reaching the capsular surface. Adjoining this tumor was another nodular lesion measuring 8 × 9 × 9 cm with lobulated grey white cut surface (Figure 1). Also seen were few smaller nodules measuring 2–4 cm in maximum dimension with brown to grey-brown cut surface. The adjacent liver parenchyma grossly appeared normal.

**Figure 2:**
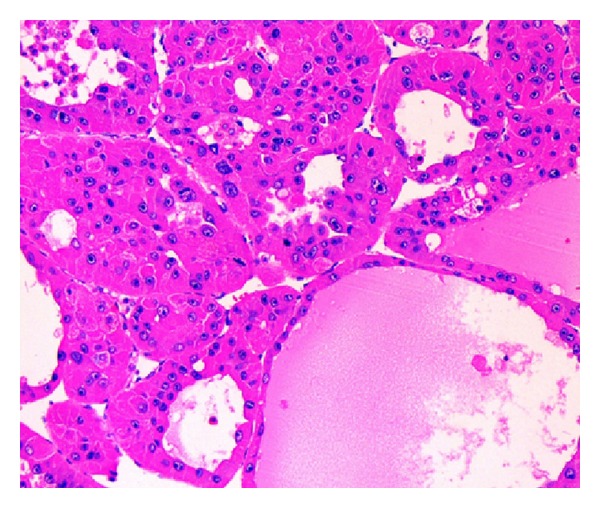
HCC showing tumor cells arranged in pseudoglandular and trabecular pattern, hematoxylin-eosin 200x.

**Figure 3:**
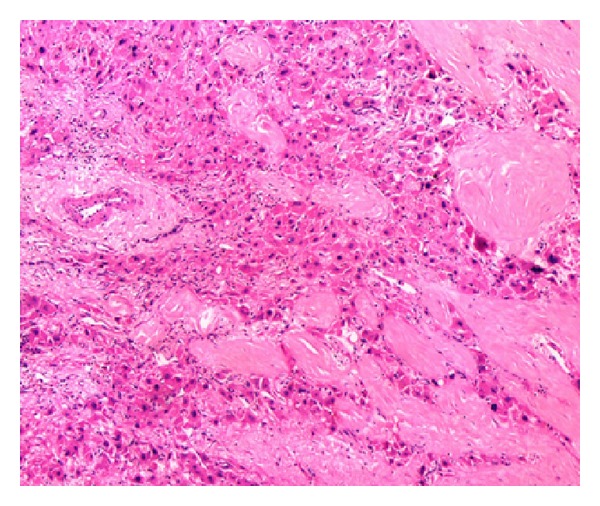
Fibrolamellar carcinoma showing tumor cells with abundant eosinophilic cytoplasm separated by lamellar bands of hyalinised collagen, hematoxylin-eosin 100x.

Microscopic examination of the larger brown tumor and the small nodules showed large polygonal cells arranged in pseudoglandular and trabecular pattern (Figure 2) separated by sinusoidal vascular channels. The tumor cells had abundant amounts of eosinophilic to amphophilic cytoplasm and pleomorphic vesicular nuclei displaying prominent nucleoli, increased mitotic activity (8-9/10 hpf) including atypical forms, and nuclear pseudoinclusions. A rim of fibrous tissue was noted around the large tumor separating it from adjacent liver in areas. Sections from the grey white mass showed a tumor with a fibrolamellar pattern composed of trabeculae of large polygonal cells separated by lamellar bands of hyalinized collagen (Figure 3). These tumor cells had abundant brightly eosinophilic granular cytoplasm with moderately pleomorphic, hyperchromatic nuclei, and some with prominent nucleoli. Eosinophilic cytoplasmic globules were present in some cells. Mitotic activity was inconspicuous. Occasional lymphovascular tumor emboli were seen in the adjacent fibrous tissue. The adjacent liver parenchyma showed preserved architecture, focal mild steatosis, and no evidence of fibrosis or cirrhosis.

**Figure 4:**
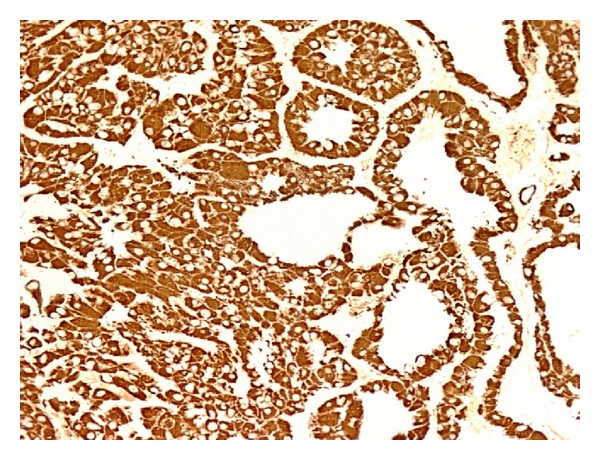
Tumor cells showing diffuse granular cytoplasmic positivity for Hep-par 1 in HCC, immunohistochemistry 200x.

**Figure 5:**
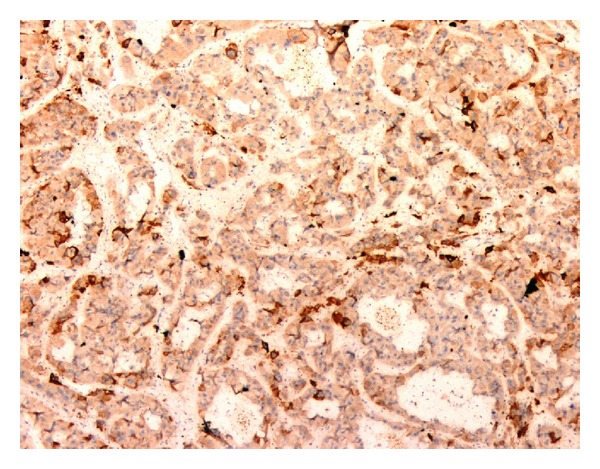
Tumor cells of HCC showing focal positivity for CK7, immunohistochemistry 100x.

**Figure 6:**
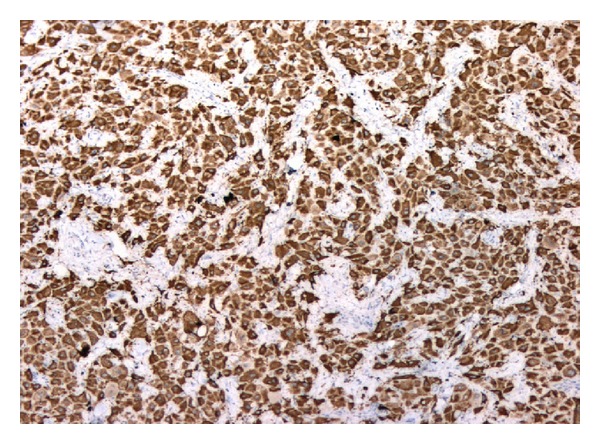
Tumor cells of FLC showing diffuse positivity for CK7, immunohistochemistry 100x.

Immunohistochemistry revealed tumor cells of HCC with diffuse positivity for Hep Par1 (Figure 4) and focal positivity for CK7 (Figure 5) whereas the tumor cells of fibrolamellar-type showed diffuse strong positivity for CK7 (Figure 6). Ep-CAM was positive in both HCC and in FLC but the percentage of positive cells and the intensity of staining were more in the HCC component. Immunostaining for beta catenin showed diffuse membranous but no nuclear staining in tumor cells of both types. A final diagnosis of multinodular combined adult type moderately differentiated HCC and FLC was made.

The patient was given chemotherapy which included cisplatin, carboplatin, and adriamycin, which he tolerated well, completed the course, and was on followup. Four months after surgery, S. bilirubin, total was 0.5 mg/dL, S. protein 7.5 g/dL, S. albumin 4.4 g/dL, AST 22 U/L, ALT 11 U/L and ALP 290 U/L.

Two years after surgery, new lesions were detected in the liver, on ultrasonography. Subsequent CT scan showed a heterogeneous mass in the porta, between the hepatic artery and inferior vena cava, fairly well-defined measuring approximately 9.7 × 4.6 × 4.6 cm with a lobulated contour and calcification on its inferior aspect, likely to represent a nodal mass. Considering disease recurrence and inoperability, the patient was put on Sorafenib treatment from March 2012. Presently, till the writing of this paper, he has survived and is on palliative care.

## 3. Discussion

FLC is an uncommon tumor which occurs in adolescents and young adults and is rare in the Asian population [1]. It is quite distinct from the usual adult type HCC as it occurs without predisposing chronic liver disease and is significantly different in morphology and immunohistochemical staining patterns compared to typical HCC [1, 6, 7]. FLC, therefore, has now been classified as a separate tumor in the latest WHO classification, hence should be analyzed separately from typical HCC [1], while earlier it was recognized as a unique histological pattern of HCC. 

There are occasional reports of FLC coexistent with or admixed with HCC [2, 4, 5]. Transformation of FLC to HCC in recurrent lesions [8, 9] has also been reported in adults, in the literature, from other countries.

We report the first case of synchronous FLC and HCC, presenting as 2 adjacent, but separate, well-defined gross and microscopic lesions in a 14-year-old boy from India. 

Typical HCC with fibrous stroma can resemble FLC but these cases should not be mistaken for mixed HCC and FLC. Immunohistochemistry is useful in resolving these cases. The tumor cells of FLC show strong positivity for CK7, which is focal in HCC, as seen in our case. A recent study has shown strong positivity for CD68 in FLC with 96% sensitivity and 80% specificity, with 98% negative predictive value [10]. These authors suggest that in addition to the typical histological features, all cases of FLC should be immunostained with CK7 and CD68 and in the absence of CK7 and/or CD68 positivity, the diagnosis of FLC should be questioned [10, 11]. Positive staining for Ep-CAM has been shown in FLC [7], which was also seen in our case.

Mutations in the beta catenin gene have been reported in all categories of hepatocellular neoplasm but not in FLC and there was no nuclear staining for beta catenin by immunohistochemistry [11]. We did not find nuclear staining for beta catenin in tumor cells of both HCC and FLC in the present case. Our previous study [12] has also shown that the beta-catenin pathway appears to be infrequent in hepatitis B related HCC in India. 

Most of the earlier case reports [2–4, 8, 9] have not performed these IHC stains and thus cannot be included for definitive comparison in retrospect. Hyaline bodies (cytoplasmic inclusions that are eosinophilic and tend to be smaller than pale bodies) are also present in nearly half of FLC cases [13]. But pale bodies and hyaline bodies are not unique to FLC, as they can also be found in ordinary HCC, so that a diagnosis of FLC should not be made on the presence of these inclusions alone [11]. In our case, on immunostaining, the tumor cells of the HCC type showed only focal positivity for CK7, whereas those of the fibrolamellar-type showed diffuse strong positivity. CD68 staining was not carried out, since the literature on that came out only much later. 

The presence of CK7 and/or CK19 has been suggested to represent evidence of hepatic progenitor cell origin [14]. The cell of origin of FLC is not certain but combined occurrence of FLC-HCC can suggest a possible derivation from hepatic progenitor cells with transdifferentiation in a subset to typical HCC phenotype and loss of CK7 positivity. 

AFP levels are typically normal in FLC [7]. So raised serum AFP levels, older age at diagnosis, and presence of chronic liver disease should deter one from making a diagnosis of FLC-HCC and such cases are best regarded as HCC for management. We found only one case of combined FLC-HCC available in the indexed English literature from Europe [5] that was comparable to our case. Currently available experience about combined FLC-HCC cases is very limited in the literature as regards their pathogenesis, morphology, disease course, or survival outcome. Ours is perhaps the first clinico-pathologically well-characterized case from Asia with treatment and follow-up details. Pure FLC is generally accepted to have an overall better prognosis than to those with HCC [11, 13]. Clinically, our patient responded well to treatment by partial hepatectomy and chemotherapy and has been doing well on followup. After two years from initial diagnosis and treatment he has recently developed recurrence in liver at the porta, which was inoperable. He has therefore been put on Sorafenib treatment and is undergoing palliative care.

The occurrence of two morphologically distinct types of liver tumor in the same patient raises the possibility that they could have arisen from prehepatocytic cancer stem cell. This may be worth investigating further in the future.
